# Effectiveness of seasonal mRNA COVID-19 vaccination against post COVID-19 condition between July 2023 and September 2024 among adults aged ≥ 60 years in Germany: a population-based cohort study

**DOI:** 10.1186/s12879-026-13803-8

**Published:** 2026-06-13

**Authors:** Nita Perumal, Caroline Peine, Annika Steffen, Ole Wichmann, Thomas Harder

**Affiliations:** Immunization Unit, Department of Infectious Disease Epidemiology, Robert Koch Institute, Seestrasse 10, 13353 Berlin, Germany

**Keywords:** COVID-19, Long COVID, Immunisation, Vaccine effectiveness, Cohort study, Germany

## Abstract

**Background:**

Some individuals infected with severe acute respiratory syndrome coronavirus 2 experience long-term symptoms, termed post COVID-19 condition (PCC). Pandemic-era studies have demonstrated moderate effectiveness of COVID-19-vaccines in preventing PCC. However, as population immunity has evolved and variant-adapted vaccines have been introduced, estimates of vaccine effectiveness and disease frequency from the post-pandemic era are needed to guide policy-making and communication.

**Methods:**

We investigated whether one mRNA COVID-19 vaccine dose during the 2023/2024 season was associated with reduced PCC risk in the following six months among adults aged ≥ 60 years in Germany. We conducted a retrospective cohort study using nationwide, outpatient claims data and performed descriptive and Poisson regression analyses.

**Results:**

In our cohort of 19,121,674 patients, 3,437,701 (18.0%) received one dose of a seasonal COVID-19 vaccine and 36,830 fulfilled the PCC case definition (incidence: 0.2%). Vaccinated individuals were older (median age 75 years) compared to those who were not vaccinated (71 years), with a higher proportion being female (54% vs. 46% male). 0.08% of vaccinated patients were diagnosed with PCC during study follow-up, compared to 0.22% non-vaccinated. One vaccine dose was associated with lower risk (adjusted Risk Ratio 0.37; 95% CI 0.36–0.39) of receiving a PCC diagnosis at six months post-vaccination compared to no vaccination, translating to a vaccine effectiveness of 63%.

**Conclusion:**

Our results demonstrate a low PCC-incidence and a strong protective association between seasonal COVID-19 vaccination and PCC during the first post-pandemic season. These findings can help improve acceptance of COVID-19-vaccines and support doctors’ and patients’ decision-making regarding vaccination.

**Supplementary information:**

The online version contains supplementary material available at 10.1186/s12879-026-13803-8.

## Background

A small percentage of individuals infected with severe acute respiratory syndrome coronavirus 2 (SARS-CoV-2) experience long-term symptoms [1, 2], which are collectively termed post COVID-19 condition (PCC). The World Health Organization defines PCC as continuation or development of new symptoms three months after the initial SARS-CoV-2 infection, with these symptoms lasting for at least two months with no other explanation [3]. The reported PCC prevalence range is large, ranging from less than 1% to 20% [1, 2]. A 2023 United Kingdom (UK) report based on self-reported symptoms estimated that 2.9% of the British population experienced long-term symptoms [4]. However, two-thirds of the patients surveyed reported contracting their infection prior to the predominance of the SARS-CoV-2 omicron variant.

Germany has a national recommendation for seasonal COVID-19-vaccination in autumn for individuals aged ≥ 60 years and for those with an elevated risk of severe COVID-19 disease [5]. The primary aim of this recommendation is prevention of severe COVID-19 disease. However, despite a continued higher risk for severe outcomes, especially among the elderly and immunocompromised patients, uptake of seasonal COVID-19 vaccines in Germany is low and declined from 21% in the 2023/2024 season to 14% in the 2024/2025 season for persons aged 60 years and older [6].

In current vaccination guidelines, as well as communications campaigns, the potential of preventing PCC with vaccines has not been explicitly addressed. A recently published systematic review showed that COVID-19 vaccines may be moderately effective in preventing PCC (effectiveness of 41% for ≥1 vaccine dose) and that vaccine effectiveness (VE) may increase with the number of vaccine doses administered [7]. However, all included studies were conducted during the pandemic. In addition, currently available evidence on the risk and prevalence of PCC is also primarily derived from the pandemic era [8]. As population immunity and COVID-19 epidemiology have changed considerably from during the pandemic to after, and as omicron variant-adapted vaccines have been introduced, data on both VE and the risk of developing PCC are needed from the post-pandemic period.

Recent studies have begun to examine the protective effect of seasonal COVID-19 vaccination and a new analysis for the autumn 2024 season in the UK demonstrated moderate VE of 43% against severe COVID-19 outcomes in the three months following vaccination [4]. However, VE of the seasonal vaccine against PCC remains to be investigated. Therefore, using real-world data for adults aged ≥ 60 years in Germany, we investigated whether a single mRNA COVID-19 vaccine dose during the autumn 2023/winter 2024 season (hereafter 2023/2024 season) was associated with a reduced risk of new-onset PCC in the following six months.

## Methods

We conducted a retrospective cohort study using nationwide outpatient claims data from all 17 Associations of Statutory Health Insurance Physicians (ASHIPs) in Germany, which have been described previously [9]. In brief, claims data for ambulatory medical services covering approximately 85% of the entire German population are submitted by physicians to the regional ASHIP on a quarterly basis and transferred into a central database. The data include basic patient information, such as birthdate, and information on all vaccinations received, as well as a large range of medical diagnoses based on International Classification of Diseases (ICD)-10 codes.

To build the study cohort, all adults aged ≥ 60 years with ≥1 documented doctor’s visit in 2021 were initially included. Our exposure of interest was a single dose of an mRNA COVID-19 vaccine (bivalent original/omicron BA.4–5 or monovalent omicron XBB.1.5 vaccines from Pfizer and Moderna) received between Quarter 3 (Q3)/2023 – Q1/2024. Our outcome of interest was the first diagnosis of PCC (ICD-10 code U09.- or U09.9, coded as being “certain”) during the follow-up period from Q4/2023 – Q3/2024 (among non-vaccinated) or, for the same follow-up period, in the six months following vaccination (among vaccinated).

Due to the association of omicron variants with milder disease [10], leading to decreased consistent testing for COVID-19 infections during our study period, we used a broader case definition and categorized individuals as PCC cases even if they did not have a documented COVID-19 diagnosis prior to their PCC diagnosis. We assumed that physicians had solid grounds for diagnosing PCC and coding it as “certain” and had ruled out other possible diagnoses. However, we conducted a sensitivity analysis in which patients were only considered to be PCC cases if they also had a preceding COVID-19 diagnosis (ICD-10 code U07.1 or U07.2).

Patients were excluded from the cohort if they had received COVID-19 vaccination in the two quarters prior to the exposure period or during follow-up (as a therapeutic role of vaccination following SARS-CoV-2 infection has not yet been conclusively ruled out [11, 12]); or had a PCC or COVID-19 diagnosis in the four quarters prior to the outcome period (to include new-onset cases only). To rule out loss of follow-up due to movement between ASHIP regions, death, or emigration out of Germany, patients were excluded if they had neither a PCC outcome during follow-up nor a documented doctor’s visit in the last two quarters of 2024. Lastly, patients were excluded if they had missing, implausible, or inconsistent values for age, sex, vaccine doses, or PCC diagnosis.

We extracted the following information from the database: age in 2023 (years), sex (male/female), number of relevant chronic disease diagnoses [13] prior to exposure period (ICD-10 three-character codes appearing in ≥ 3 quarters between Q1/2021 – Q2/2023, clustered into disease groups; see Additional file 1 Table S1), and history of prior PCC diagnoses between Q1/2021 – Q3/2022 (yes/no).

**Fig. 1:**
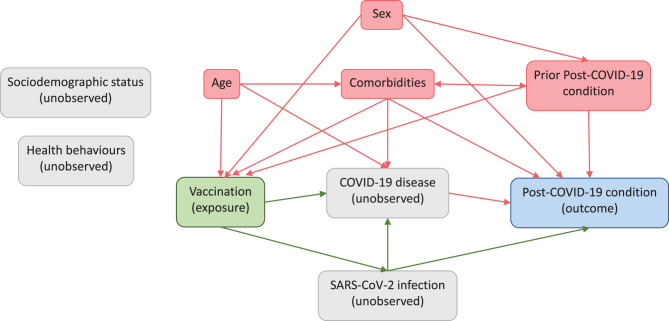
Directed acyclic graph modeling hypothesized causal relationship between seasonal COVID-19 vaccination and post COVID-19 condition

We conducted descriptive analyses by summarizing categorical data as counts and percentages and described continuous data as median values with interquartile ranges (IQRs). We created a directed acyclic graph in DAGitty [14] that modeled our hypothesized causal relationship between vaccination and PCC (Fig. 1) and identified a minimum adjustment set for observable variables (age, sex, prior PCC status, and number of chronic disease groups) to estimate Risk Ratios (RR) for our cohort study design.

Specifically, we used a generalized linear model to conduct a Poisson regression with log link and robust variance to estimate the RRs, since our outcome was rare and follow-up time was short. Our final model consisted of a binary PCC outcome variable (yes/no) as the dependent variable and vaccination status (yes/no), age (continuous), sex (male/female), prior PCC status (yes/no), and number of chronic disease groups (1, 2, 3+) as the dependent variables. Results are presented as adjusted Risk Ratios (aRR) with 95% confidence intervals (CI) and VE was calculated as (1 – aRR) x 100%. We also calculated the Number Needed to Vaccinate (NNV) to provide an absolute measure of effect for our results using the formula NNV = 1 / (PCC incidence among Unvaccinated ×VE) [15].

To ensure that prior PCC diagnosis between Q1/2021 – Q3/2022 was not an effect modifier, we also replicated the primary analysis stratified by prior PCC diagnosis status and examined the RR estimates between the two strata.

Database queries and data extraction were conducted using Microsoft SQL Server Management Studio and data cleaning and analyses were conducted in STATA Version 19.

## Results

**Fig. 2:**
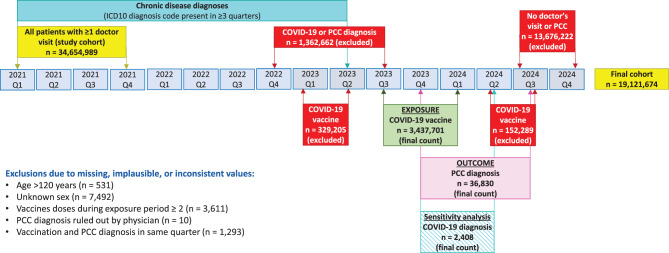
Overview of design, timeline, and inclusion/exclusion criteria of study cohort (German adults aged ≥ 60 years)

A total of 19,121,674 unique patients were included in our final cohort, of which 3,437,701 (18.0%) received one dose of an mRNA COVID-19 vaccine in the 2023/2024 season. Over the subsequent follow-up period (Q4/2023 to Q3/2024) a total of 36,830 patients were identified that fulfilled our PCC case definition, which translates into a PCC incidence of 0.2%. Figure 2 provides a detailed overview of the design, timeline, and inclusion/exclusion criteria of our study cohort, including the number of unique patient records included or excluded at each step.

The characteristics of our final study population by vaccination status are provided in Table 1 (population characteristics by outcome status are provided in Additional file 1 Table S2). Patients who received one dose of an mRNA COVID-19 vaccine in the 2023/2024 season were older (median age 75 years, IQR: 68–82) compared to those who were not vaccinated during the season (71 years, 65–79), with a higher proportion being female (54% vs. 46%). A lower proportion of vaccinated patients were diagnosed with PCC during study follow-up (0.08% vs. 0.22%) or in the preceding years (0.6% vs. 0.8%). Among those who were not vaccinated, a lower proportion had no chronic disease diagnoses in the period prior to vaccination (29% vs. 13%), whereas a higher proportion of those vaccinated had three or more chronic disease groups diagnoses in the period prior to vaccination (17% vs. 11%).

**Table 1:** Characteristics of the study population by vaccination status (single COVID-19 vaccine dose during 2023/2024 season)

Variables	Non-vaccinated		Vaccinated		Total	
	Number	%	Number	%	Number	%
**N**	15,683,973	82.0	3,437,701	18.0	19,121,674	100
**Median Age in years [IQR]**	71 [65–79]		75 [68–82]		72 [66–80]	
**Age group**						
60–69 years	6,797,697	43.3	1,047,365	30.5	7,845,062	41.0
70–79 years	4,965,330	31.7	1,208,824	35.2	6,174,154	32.3
80+ years	3,920,946	25.0	1,181,512	34.4	5,102,458	26.7
**Sex**						
Male	6,581,489	42.0	1,583,810	46.1	8,165,299	42.7
Female	9,102,484	58.0	1,853,891	53.9	10,956,375	57.3
**PCC diagnosis during follow-up (outcome)**						
No	15,649,750	99.78	3,435,094	99.92	19,084,844	99.8
Yes	34,223	0.22	2,607	0.08	36,830	0.2
**Prior PCC diagnosis**						
No	15,561,718	99.2	3,417,098	99.4	18,978,816	99.3
Yes	122,255	0.8	20,603	0.6	142,858	0.7
**PCC patients with COVID-19 diagnosis**						
No	15,681,607	99.98	3,437,471	99.99	19,119,078	99.99
Yes	2,366	0.02	230	0.01	2,596	0.01
**Number of chronic disease groups**^**a**^						
0	4,469,449	28.5	458,201	13.3	4,927,650	25.8
1	5,417,635	34.5	1,253,240	36.5	6,670,875	34.9
2	4,104,399	26.2	1,158,584	33.7	5,262,983	27.5
3+	1,692,490	10.8	567,676	16.5	2,260,166	11.8

IQR = Interquartile range; PCC = Post COVID-19 condition

^a^ HIV, cancer, immune system, nervous system, endocrinal/metabolic/diabetes, cardiovascular system, respiratory system, gastrointestinal system, liver disease, musculoskeletal/rheumatoid diagnoses

Among all patients, 2,596 (0.01%) had a preceding documented COVID-19 diagnosis, irrespective of vaccination status. When restricting the PCC patients to only those with a preceding COVID-19 diagnosis for the sensitivity analysis, 2,366 non-vaccinated patients received a PCC diagnosis during study follow-up, in comparison to 42 vaccinated patients, with 188 patients being excluded as their vaccinations occurred in the same quarter as their COVID-19 diagnoses or thereafter. Additional file 1 Tables S3 and S4 provide an overview of the characteristics of the study population included in the sensitivity analysis.

Table 2 presents the crude and adjusted RR estimates from the Poisson regression analyses for the primary and sensitivity analyses. Among the primary study population of German adults aged ≥ 60 years, a single dose of the mRNA COVID-19 seasonal vaccine was associated, on average, with lower risk (aRR 0.37; 95% CI 0.36–0.39) of receiving a PCC diagnosis in the following six months compared to no seasonal vaccination, adjusted for age, sex, prior PCC status, and number of chronic diseases groups. This translates to a VE of 63% (95% CI 61–64), corresponding to an NNV of 7.2 (95% CI 7.1–7.5). The aRR was lower in the sensitivity analysis (aRR 0.09; 95% CI 0.07–0.12), when the PCC case population was restricted to only those with a recent, preceding COVID-19 diagnosis, translating to a VE of 91% (95% CI 88–93) and a corresponding NNV of 55.0 (95% CI 53.8–56.8).

**Table 2:** Crude and adjusted risk ratios for association between mRNA COVID-19 vaccine and post COVID-19 condition

Variables	**Primary Analysis**^**b**^**(N = 19,121,674)**		**Sensitivity Analysis**^**b**^**(N = 19,087,252)**	
	PCC diagnosis outcome (all patients)		COVID-19 + PCC diagnoses outcome	
	Crude RR (95% CI)	Adjusted RR (95% CI)	Crude RR (95% CI)	Adjusted RR (95% CI)
**Vaccinated, 1 dose** **(exposure of interest)**				
No	Reference	Reference	Reference	Reference
Yes	0.35 (0.33, 0.36)	0.37 (0.36, 0.39)	0.08 (0.06, 0.11)	0.09 (0.07, 0.12)
**Age**	0.95 (0.95, 0.95)	0.96 (0.95, 0.96)	0.93 (0.92, 0.93)	0.93 (0.92, 0.93)
**Sex**				
Male	Reference	Reference	Reference	Reference
Female	1.36 (1.33, 1.39)	1.40 (1.37, 1.43)	1.57 (1.44, 1.71)	1.66 (1.52, 1.81)
**Prior PCC diagnosis**				
No	Reference	Reference	Reference	Reference
Yes	18.35 (17.79, 18.93)	14.54 (14.08, 15.00)	9.41 (8.00, 11.07)	6.71 (5.70, 7.91)
**Number of chronic disease groups**^**a**^				
0	Reference	Reference	Reference	Reference
1	1.24 (1.20, 1.27)	1.39 (1.35, 1.43)	1.18 (1.06, 1.32)	1.46 (1.31, 1.63)
2	1.30 (1.27, 1.34)	1.62 (1.57, 1.67)	1.28 (1.14, 1.43)	1.87 (1.67, 2.09)
3+	1.69 (1.64, 1.75)	2.22 (2.14, 2.30)	1.56 (1.36, 1.78)	2.55 (2.23, 2.91)

CI = confidence interval; RR = Risk Ratio; PCC = Post COVID-19 condition

^a^ HIV, cancer, immune system, nervous system, endocrinal/metabolic/diabetes, cardiovascular system, respiratory system, gastrointestinal system, liver, musculoskeletal/rheumatoid

^b^ Final model consisted of a binary PCC outcome variable (yes/no) as the dependent variable and vaccination status (yes/no), age (continuous), sex (male/female), prior PCC status (yes/no), and number of chronic disease groups (1, 2, 3+) as the dependent variables

When the primary analysis was repeated by stratifying the study population by prior PCC diagnosis status, the RR among those with prior PCC was 0.42 (95% CI 0.37–0.47) and RR among those without prior PCC was 0.37 (95% CI 0.35–0.38).

## Discussion

To our knowledge, this is the first study to investigate the association between the omicron variant-adapted COVID-19 vaccines and subsequent PCC. Although a large body of literature exists on the protective effect of the pandemic-era vaccines in preventing PCC [7, 16], data on the post-pandemic variant-adapted seasonal vaccines is scarce. The incidence of PCC in our cohort for the 2023/2024 season was 0.2%, similar to recent estimates from Netherlands [1], and lower than the PCC prevalence estimates reported from pandemic-era studies [17]. This indicates that a number of factors, such as higher population-level immunity to SARS-CoV-2, changing characteristics of the circulating variants, and an evolving risk perception of COVID-19 among patients and physicians, might have contributed towards a new post-pandemic paradigm of PCC. Thus, the results of our study fill a critical gap in knowledge regarding the potential benefits of the omicron variant-adapted seasonal COVID-19 vaccine and demonstrate that it may not only continue to protect against severe COVID-19 outcomes, but also against PCC. Given the low seasonal COVID-19 vaccination coverage in many European countries (including Germany) [18, 19], our results are timely and pertinent as they can support doctors’ and patients’ decision-making in the current season, provide important evidence to technical advisory committees regarding vaccination guidelines, as well as inform communications campaigns for upcoming seasons.

The VE estimates in our study are in line with previous population-based cohort studies [16, 20], but higher than the results of a comprehensive recent systematic review that reported that vaccination prior to infection confers a moderate level of protection against PCC, with a VE of 41% [7]. However, only a few studies included in the review reported on infections during omicron predominance and the protection of seasonal vaccination was not analyzed at all. Furthermore, most studies conducted so far have investigated the association between vaccination and PCC among COVID-19 patients only. In contrast, our study examined the potential real-world, total effect of vaccination on PCC, which includes the effect of vaccination in preventing an initial SARS-CoV-2 infection, as well as in preventing symptomatic COVID-19 disease or attenuating severe outcomes following infection. We further calculated the NNV for our study period and population, which can provide guidance to public health decision makers and researchers on the absolute impact of vaccination versus its economic costs. Nonetheless, by excluding patients with vaccinations in the two quarters before our exposure period and during follow-up, as well as patients with COVID-19 and/or PCC diagnoses in the year prior, we may have selected our study cohort to be healthier or at lower risk for repeated SARS-CoV-2 infections and, therefore, at lower risk of PCC.

Our sensitivity analysis investigated the robustness of the association between vaccination and PCC by restricting patients to only those with a preceding COVID-19 diagnosis, which yielded an even higher VE. This may be partially attributable to the effectiveness of vaccination in preventing severe outcomes (followed by long-term symptoms) among our study population of adults aged ≥ 60 years [4], in addition to its protective effect against PCC. However, another possible explanation may be the nature of our data source, which only encompasses data from ambulatory care settings. Patients with a COVID-19 diagnosis during our study period are either likely to have been highly symptomatic or be at high-risk (e.g. being over 80 years of age) in order to have been tested by their physicians. Our data does not contain information on hospitalizations that may have occurred in case of severe COVID-19 disease or whether PCC was diagnosed in a hospital-care setting thereafter, nor do they contain other outcomes following severe disease, such as death. PCC symptoms also can overlap with symptoms from other old-age related conditions in our cohort, making it difficult for physicians to rule out other diagnoses, which could explain the protective effect of older age found in our and other studies [13]. On the other hand, a different reason for a COVID-19 diagnosis may be the testing of patients before elective surgeries, a requirement that was common during the pandemic and may still be implemented in certain settings. In this case, patients may be asymptomatic and relatively healthy, with lower risk for PCC, but may still have a COVID-19 diagnosis in their records.

Having a history of PCC was very strongly associated with being re-diagnosed with PCC during our study period, although patients with any PCC diagnosis in the year prior to our exposure period were excluded. This may in part be due to individual immunological characteristics, as well as the long-term organ damage and other systemic changes attributed to PCC [21], which may predispose patients who have once experienced PCC to future PCC episodes [22]. This may be especially relevant in the current landscape of rapidly evolving Omicron variants, which generally spread more easily and are more immune-evasive than previous variants [23, 24], thereby increasing the risk of reinfections. Physicians may also be more likely to diagnose PCC again in patients who have a prior history of PCC, due to the patients’ (and their physicians’) familiarity with the symptomology and disease course. To rule out effect modification by prior PCC status, we conducted additional analyses by stratifying our study population by prior PCC diagnosis status The RR point estimates of the primary analysis changed minimally, with 95% CIs that overlapped, indicating that prior PCC diagnosis was not an important effect modifier in our model.

Our study has several strengths. Using real-world data encompassing 85% of the general German population aged ≥ 60 years, we built a cohort of over 19 million unique individuals, where our outcome was a reliable medical diagnosis of PCC, rather than a self-reported measure. Similarly, our exposure variable was based on reliable insurance claims data and not on self-reported vaccinations. To our knowledge, this is the first study to investigate the total effect of vaccination on PCC in the general population, rather than among COVID-19 patients only, and is also the first to report on VE of the seasonal COVID-19 vaccine against PCC, which is the new norm for COVID-19 prevention going forward. Our study also provides updated information on PCC incidence in the post-pandemic era and demonstrates that, although PCC continues to be a challenge among the general population, it is being diagnosed with less frequency in comparison to during the pandemic period. Hence, the results presented here are not only generalizable, but also current and relevant, and allow for clearer communication of the overall benefits of seasonal COVID-19 vaccination among older adults.

Nonetheless, our study also has a number of limitations. We rely on secondary data; thus, our dataset may contain coding errors and misclassifications. Although more reliable than self-report, even a medical diagnosis of PCC is still likely to be subjective and variable between physicians, due to PCC’s broad and heterogeneous nature, and PCC may be misclassified in our cohort. Similarly, our sensitivity analysis relies on COVID-19 diagnosis data and not on COVID-19 laboratory test data. Our dataset contains only broad time categories (quarters), lacking precise dates, and does not include information from the hospital and private-care sector or information on patients’ health behaviours and socioeconomic status. Although we use the number of chronic disease groups previously diagnosed as a proxy for health status, this probably does not sufficiently adjust for the confounding or mediating effects of the numerous patient-level factors, such as number of doctor’s visits, that influence COVID-19 vaccine uptake [25] or PCC risk. Vaccinated individuals in our cohort also had a higher burden of chronic diseases than not vaccinated, which could lead to PCC being underdiagnosed among the vaccinated due to overlapping symptoms. Therefore, our results could be overestimating VE against PCC and should be taken as an indication of the likely benefit of seasonal COVID-19 vaccination against PCC, with further robust analyses required to corroborate the extent of this benefit.

## Conclusion

Using real-world data, our study examines the relationship between COVID-19 vaccination in the 2023/2024 season with having a PCC diagnosis in the following six months among ≥ 60-year-olds in Germany and demonstrates a strong protective association. Our results also indicate that incidence of PCC during the first post-pandemic season was low in Germany. These findings can help improve acceptance of COVID-19-vaccines and support doctors’ and patients’ decision-making regarding vaccination in the upcoming season. Furthermore, our results can provide important evidence to technical advisory committees regarding vaccination guidelines, as well as inform future communications campaigns.

## Electronic supplementary material

Below is the link to the electronic supplementary material.

Supplementary Material 1

## Data Availability

Individual-level data cannot be made publicly available due to German and European Union data protection regulations. However, all the data used for this analysis are included as aggregated data in the manuscript tables and in Additional file 1.

## Abbreviations

SARS-CoV-2: Severe acute respiratory syndrome coronavirus 2
PCC: Post COVID-19 condition
UK: United Kingdom
VE: Vaccine effectiveness
ASHIPs: Associations of Statutory Health Insurance Physicians
ICD: International Classification of Diseases
IQR: Interquartile range
RR: Risk Ratio
CI: Confidence intervals
Q: Quarter
