# Supplementary material for: Effectiveness of seasonal mRNA COVID-19 vaccination against post COVID-19 condition between July 2023 and September 2024 among adults aged ≥ 60 years in Germany: a population-based cohort study
Source: BMC Infect Dis. 2026 Jun 13;26:1140. doi: 10.1186/s12879-026-13803-8 (PMC13264817; doi:10.1186/s12879-026-13803-8)
Supplement: Supplementary file 1 — Supplementary Material 1 [file 12879_2026_13803_MOESM1_ESM.docx]

**Table S1:** List of International Classification of Diseases (ICD)-10 codes extracted from outpatient claims data to build chronic disease groups for analysis.

| **ICD-10 codes** | **Disease group (ENG)** |
| --- | --- |
| B20, B21, B22, B23, B24 | HIV |
| C*, C00, C01, C02, C03, C04, C05, C06, C07, C08, C09, C10, C11, C12, C13, C14, C21, C32, C51, C52, C53, C54, C60, C81, C82, C83, C84, C85, C86, C88, C90, C91, C92, C93, C94, C95, C96, C97, C97! | Cancer |
| D57, D68, D69, D70, D71, D72, D73, "D73.0", D75, D76, D77, D77*, D80, D81, D82, D83, D84, D85, D86, D89, D90 | Immune system |
| E05, E06, E10.-, E11.-, E12.-, E13.-, E14.-, E10, E11, E12, E13, E14, E66 | Endocrinal system, Metabolic diseases, Diabetes |
| G35, G61, G70, G93 | Nervous system |
| I09, I10, I11, I12, I13, I15, I20, I21, I22, I24, I25, I42, I43, I48, I50, I67 | Cardiovascular system |
| J44, J45, | Respiratory system |
| K50, K51, | Gastrointestinal system |
| B18, K72, K73, K74 | Liver conditions |
| M05, M06, M08, M31, M32, M35 | Musculoskeletal system, Rheumatoid arthritis |

**Table S2:** Characteristics of primary study population (German adults aged ≥60 years) by Post COVID-19 condition status during follow-up.

| **Variables** | **No PCC diagnosis** | | **PCC diagnosis** | | **All** | |
| --- | --- | --- | --- | --- | --- | --- |
|  | **Number** | **%** | **Number** | **%** | **Number** | **%** |
| **N** | 19,084,844 | 99.8 | 36,830 | 0.2 | 19,121,674 | 100 |
| **Median Age in years [IQR]** | 72 [66-80] | | 68 [63-76] | | 72 [66-80] | |
| **Age group** |  |  |  |  |  |  |
| 60-69 | 7,824,452 | 41.0 | 20,610 | 56.0 | 7,845,062 | 41.0 |
| 70-79 | 6,164,171 | 32.3 | 9,983 | 27.1 | 6,174,154 | 32.3 |
| 80+ | 5,096,221 | 26.7 | 6,237 | 16.9 | 5,102,458 | 26.7 |
| **Sex** |  |  |  |  |  |  |
| Male | 8,152,263 | 42.7 | 13,036 | 35.4 | 8,165,299 | 42.7 |
| Female | 10,932,581 | 57.3 | 23,794 | 64.6 | 10,956,375 | 57.3 |
| **Vaccinated, 1 dose (exposure)** |  |  |  |  |  |  |
| No | 15,649,750 | 82.0 | 34,223 | 92.9 | 15,683,973 | 82.0 |
| Yes | 3,435,094 | 18.0 | 2,607 | 7.1 | 3,437,701 | 18.0 |
| **Prior PCC diagnosis** |  |  |  |  |  |  |
| No | 18,946,456 | 99.3 | 32,360 | 87.9 | 18,978,816 | 99.3 |
| Yes | 138,388 | 0.7 | 4,470 | 12.1 | 142,858 | 0.7 |
| **PCC patients with prior COVID-19 diagnosis** |  |  |  |  |  |  |
| No | 19,084,844 | 100.0 | 34,234 | 93.0 | 19,119,078 | 99.9 |
| Yes | NA | -- | 2,596 | 7.0 | 2,596 | 0.01 |
| **Number of chronic disease groups*** |  |  |  |  |  |  |
| 0 | 4,920,046 | 25.8 | 7,604 | 20.6 | 4,927,650 | 25.8 |
| 1 | 6,658,132 | 34.9 | 12,743 | 34.6 | 6,670,875 | 34.9 |
| 2 | 5,252,400 | 27.5 | 10,583 | 28.7 | 5,262,983 | 27.5 |
| 3+ | 2,254,266 | 11.8 | 5,900 | 16.0 | 2,260,166 | 11.8 |

IQR=Interquartile range; PCC=Post COVID-19 condition

* HIV, cancer, immune system, nervous system, endocrinal/metabolic/diabetes, cardiovascular system, respiratory system, gastrointestinal system, liver, musculoskeletal/rheumatoid

**Table S3:** Characteristics of study population included in sensitivity analysis (PCC cases with preceding COVID-19 diagnosis only) - German adults aged ≥60 years by vaccination status.

| **Variables** | **Non-vaccinated** | | **Vaccinated** | | **All** | |
| --- | --- | --- | --- | --- | --- | --- |
|  | **Number** | **%** | **Number** | **%** | **Number** | **%** |
| **N** | 15,652,116 | 82.0 | 3,435,136 | 18.0 | 19,087,252 | 100 |
| **Median Age in years [IQR]** | 71 [65-80] | | 75 [68-82] | | 72 [66-80] | |
| **Age group** |  |  |  |  |  |  |
| 60-69 | 6,779,747 | 43.3 | 1,046,254 | 30.5 | 7,826,001 | 41.0 |
| 70-79 | 4,956,654 | 31.7 | 1,207,975 | 35.2 | 6,164,629 | 32.3 |
| 80+ | 3,915,715 | 25.0 | 1,180,907 | 34.4 | 5,096,622 | 26.7 |
| **Sex** |  |  |  |  |  |  |
| Male | 6,570,334 | 42.0 | 1,582,705 | 46.1 | 8,153,039 | 42.7 |
| Female | 9,081,782 | 58.0 | 1,852,431 | 53.9 | 10,934,213 | 57.3 |
| **PCC diagnosis during follow-up (outcome)** |  |  |  |  |  |  |
| No | 15,649,750 | 99.98 | 3,435,094 | 100 | 19,084,844 | 99.99 |
| Yes | 2,366 | 0.02 | 42 | 0.00 | 2,408 | 0.01 |
| **Prior PCC diagnosis** |  |  |  |  |  |  |
| No | 15,533,906 | 99.2 | 3,414,803 | 99.4 | 18,948,709 | 99.3 |
| Yes | 118,210 | 0.8 | 20,333 | 0.6 | 138,543 | 0.7 |
| **Number of chronic disease groups*** |  |  |  |  |  |  |
| 0 | 4,462,730 | 28.5 | 457,831 | 13.3 | 4,920,561 | 25.8 |
| 1 | 5,406,520 | 34.5 | 1,252,434 | 36.5 | 6,658,954 | 34.9 |
| 2 | 4,095,378 | 26.2 | 1,157,726 | 33.7 | 5,253,104 | 27.5 |
| 3+ | 1,687,488 | 10.8 | 567,145 | 16.5 | 2,254,633 | 11.8 |

IQR=Interquartile range; PCC=Post COVID-19 condition

* HIV, cancer, immune system, nervous system, endocrinal/metabolic/diabetes, cardiovascular system, respiratory system, gastrointestinal system, liver, musculoskeletal/rheumatoid

**Table S4:** Characteristics of study population included in sensitivity analysis (PCC cases with preceding COVID-19 diagnosis only) - German adults aged ≥60 years by Post COVID-19 condition status during follow-up.

| **Variables** | **No PCC diagnosis** | | **PCC diagnosis** | | **All** | |
| --- | --- | --- | --- | --- | --- | --- |
|  | **Number** | **%** | **Number** | **%** | **Number** | **%** |
| **N** | 19,084,844 | 99.99 | 2,408 | 0.01 | 19,087,252 | 100.0 |
| **Median Age in years [IQR]** | 72 [66-80] | | 64 [61-74] | | 72 [66-80] | |
| **Age group** |  |  |  |  |  |  |
| 60-69 | 7,824,452 | 41.0 | 1,549 | 64.3 | 7,826,001 | 41.0 |
| 70-79 | 6,164,171 | 32.3 | 458 | 19.0 | 6,164,629 | 32.3 |
| 80+ | 5,096,221 | 26.7 | 401 | 16.7 | 5,096,622 | 26.7 |
| **Sex** |  |  |  |  |  |  |
| Male | 8,152,263 | 42.7 | 776 | 32.2 | 8,153,039 | 42.7 |
| Female | 10,932,581 | 57.3 | 1,632 | 67.8 | 10,934,213 | 57.3 |
| **Vaccinated, 1 dose (exposure)** |  |  |  |  |  |  |
| No | 15,649,750 | 82.0 | 2,366 | 98.3 | 15,652,116 | 82.0 |
| Yes | 3,435,094 | 18.0 | 42 | 1.7 | 3,435,136 | 18.0 |
| **Prior PCC diagnosis** |  |  |  |  |  |  |
| No | 18,946,456 | 99.3 | 2,253 | 93.6 | 18,948,709 | 99.3 |
| Yes | 138,388 | 0.7 | 155 | 6.4 | 138,543 | 0.7 |
| **Number of chronic disease groups*** |  |  |  |  |  |  |
| 0 | 4,920,046 | 25.8 | 515 | 21.4 | 4,920,561 | 25.8 |
| 1 | 6,658,132 | 34.9 | 822 | 34.1 | 6,658,954 | 34.9 |
| 2 | 5,252,400 | 27.5 | 704 | 29.2 | 5,253,104 | 27.5 |
| 3+ | 2,254,266 | 11.8 | 367 | 15.2 | 2,254,633 | 11.8 |

IQR=Interquartile range; PCC=Post COVID-19 condition

* HIV, cancer, immune system, nervous system, endocrinal/metabolic/diabetes, cardiovascular system, respiratory system, gastrointestinal system, liver, musculoskeletal/rheumatoid
